# Screening of Ginkgo Individuals with Superior Growth Structural Characteristics in Different Genetic Groups Using Terrestrial Laser Scanning (TLS) Data

**DOI:** 10.34133/plantphenomics.0092

**Published:** 2023-09-22

**Authors:** Wen Gao, Xiaoming Yang, Lin Cao, Fuliang Cao, Hao Liu, Quan Qiu, Meng Shen, Pengfei Yu, Yuhua Liu, Xin Shen

**Affiliations:** ^1^ Co-Innovation Center for Sustainable Forestry in Southern China, Nanjing Forestry University, Nanjing, Jiangsu 210037, PR China.; ^2^ College of Forestry and Landscape Architecture, South China Agricultural University, Guangzhou, Guangdong 510642, PR China.; ^3^ Suining County Runqi Investment Co. Ltd., Xuzhou, Jiangsu 221200, PR China.; ^4^ Jiangsu Vocational College of Agriculture and Forestry, Zhenjiang, Jiangsu 212400, PR China.

## Abstract

With the concept of sustainable management of plantations, individual trees with excellent characteristics in plantations have received attention from breeders. To improve and maintain long-term productivity, accurate and high-throughput access to phenotypic characteristics is essential when establishing breeding strategies. Meanwhile, genetic diversity is also an important issue that must be considered, especially for plantations without seed source information. This study was carried out in a ginkgo timber plantation. We used simple sequence repeat (SSR) markers for genetic background analysis and high-density terrestrial laser scanning for growth structural characteristic extraction, aiming to provide a possibility of applying remote sensing approaches for forest breeding. First, we analyzed the genetic diversity and population structure, and grouped individual trees according to the genetic distance. Then, the growth structural characteristics (height, diameter at breast height, crown width, crown area, crown volume, height to living crown, trunk volume, biomass of all components) were extracted. Finally, individual trees in each group were comprehensively evaluated and the best-performing ones were selected. Results illustrate that terrestrial laser scanning (TLS) point cloud data can provide nondestructive estimates of the growth structural characteristics at fine scale. From the ginkgo plantation containing high genetic diversity (average polymorphism information content index was 0.719) and high variation in growth structural characteristics (coefficient of variation ranged from 21.822% to 85.477%), 11 excellent individual trees with superior growth were determined. Our study guides the scientific management of plantations and also provides a potential for applying remote sensing technologies to accelerate forest breeding.

## Graphical abstract

**Figure:**
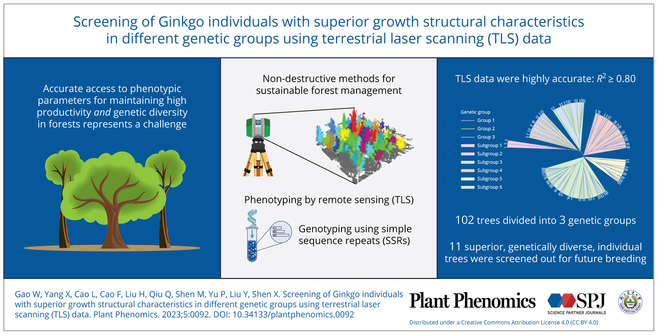

## Introduction

Forests are the largest, most widespread, and most complex terrestrial ecosystems [1]. Not only are forests the largest carbon sinks in terrestrial ecosystems [2], they also play an important role in maintaining ecological balance and improving the environment [3]. Plantations are among the most important components of the forest ecosystem, providing a wide range of benefits for society and the economy [4]. Ginkgo (*Ginkgo biloba* L.), which has edible, medicinal, timber, and landscaping functions [5], is commonly cultivated worldwide [6]. It is one of the main plantation species in China [7], widely planted in Jiangsu, Anhui, Zhejiang, and other provinces [8]. Ginkgo has a long history of cultivation [9], and breeders have gradually cultivated more than a hundred excellent varieties.

In recent years, with the concept of sustainable management of plantations [10], a large number of individual trees with excellent characteristics in plantations have received attention from breeders. Short-term breeding is shifting to long-term breeding aiming to maintain an optimal balance between genetic diversity and continuous genetic gain [11]. China possesses the world's largest afforestation land area [12], but many of them have lost information on their seed sources, making their genetic background unknown. Selection of breeding material based on phenotypes only and ignoring the genetic background may result in breeding populations with low levels of genetic diversity. Genetic diversity can show the richness of population variation and its adaptability to the environment [12], and is increasingly used in the forest breeding, forest survey, and forest resource conservation. It is necessary to consider both phenotypic superiority and genetic diversity. Simple sequence repeats (SSRs), which have the characteristics of abundant number, strong polymorphism, wide distribution, and co-dominant inheritance [13], are widely used in genetic diversity analysis of woody plants, such as chestnut [14], spruce [15], poplar [16], and also ginkgo [17].

Usually, the whole process of forest breeding is time consuming, with the genetic variation being evaluated in multiple repeated selections [18]. To accelerate the breeding process, accurate and high-throughput access to quantitative data is essential [19]. Traditional phenotype acquisition has limitations of being labor intensive, destructive, time consuming, and inefficient, making it difficult to meet the needs of large-scale work [20,21]. Using remote sensing technology, we can obtain multi-temporal, multi-scale, and multi-dimensional data of forests, thus overcoming the limitations of traditional phenotype acquisition [22]. Advanced remote sensing techniques have recently provided effective tools for estimating phenotypic characteristics [23]. As a mature and promising remote sensing technique, light detection and ranging (LiDAR) can extract the distances between the sensor and targets by tracking time intervals to provide 3-dimensional (3D) structure information about forests [24]. It has been proven as an efficient method for the high-accuracy and nondestructive measurement of various forest structure parameters [25]. Terrestrial laser scanning (TLS), alternatively terrestrial LiDAR, obtains high-density point cloud data with accuracy up to millimeter level [26,27]. Compared to airborne laser scanning (ALS), TLS can better obtain the trunk and ground features [28]. The application of the TLS enables the nondestructive estimate of tree trunk profile, as well as tree branching structure, which enables a better estimation of growth structural characteristics [16]. Growth structural characteristics such as leaf area index, trunk volume, diameter at breast height, crown width, height, and biomass can be extracted directly or indirectly (e.g., inverse models), providing an in-depth understanding of tree growth and response to different environments [29,30]. With the concept of sustainable management of plantations and the lack of seed source information in a large number of plantations in China, how to efficiently and rapidly screen superior trees in plantations and retain a certain degree of genetic diversity in the population has become a problem. As far as we know, there are few studies using point cloud data to extract traits for breeding. Moreover, high-throughput phenotype extraction combined with the genetic analysis of ginkgo species for superior individual tree screening is seldom seen in the literatures.

Our study subject is a ginkgo timber plantation that lost seed source information. This study demonstrates a practical example of superior tree screening that combines genetic analysis and high-throughput phenotypic extraction. Individual trees were grouped according to the genetic diversity and population structure analysis based on SSR. Meanwhile, growth structural characteristics were extracted using TLS data (of high-density point clouds). Comprehensive evaluation was performed by principal components analysis (PCA) of growth structural characteristics to screen for superior individual trees in each genetic group. The specific objectives are (a) to analyze the genetic diversity and population structure of the Ginkgo plantation so that individual trees can be grouped; (b) to use 3D point cloud data for individual tree growth structural characteristic extraction, thereby verifying the availability of TLS point cloud data in quantifying individual tree growth; and (c) to select superior individual trees with the best growth and preserve genetic diversity as much as possible, thus providing breeding materials for sustainable utilization of the plantation. This study demonstrates the feasibility of terrestrial LiDAR in extracting growth structural characteristics of individual tree. In addition, this study innovatively combines genetic analysis with high-throughput phenotypic acquisition to preserve genetic diversity and phenotypic advantages at the same time. This study therefore reveals the prospect of the combination of remote sensing technology and genetic analysis, provides technical and methodological support for the sustainable management and precise cultivation of plantation, and provides enlightenment for the application of remote sensing technology to accelerate the forest breeding.

## Materials and Methods

**Fig. 1.**
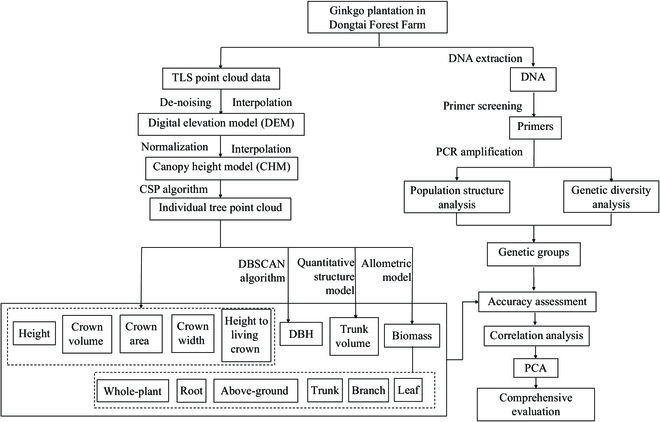
Overview of the workflow in this study.

In this study, SSR markers were used to analyze the genetic diversity and population structure of the 102 individual trees in a ginkgo timber plantation. The individual trees were divided into different genetic groups. Meanwhile, canopy height model (CHM) was derived from point clouds obtained using TLS. Height (H), crown volume (CV), and crown area (CA) of individual trees were automatically extracted according to optimal individual tree segmentation results obtained by comparative shortest-path (CSP) algorithm. The diameter at breast height (DBH) was obtained by the density-based spatial clustering of applications with noise (DBSCAN) algorithm. Crown width (CW) and height to living crown (HLC) were extracted according to the point cloud data. To obtain trunk volume (TV), we reconstructed the 3D branch geometry of the individual tree using the quantitative structure model (QSM). The biomass of all components was calculated using allometric models. Then, the accuracy of H and DBH was evaluated according to the field-measured data. Finally, best individual trees in different genetic groups were assessed using PCA. The workflow of this study is shown in Fig. 1.

### Study area description and data acquisition

The experiment site is located in the Dongtai Forest Farm (120°47′ to 120°51′E, 32°51′ to 32°53′N), which is on the eastern coast of Dongtai City, Jiangsu Province, China. The Dongtai Forest Farm experiences a mild climate, with an average annual temperature and precipitation of 14.6 °C and 1,050 mm, respectively. The farm covers 4,156.93 ha, with 85% forest cover, mainly planted with *G. biloba*, *Metasequoia glyptostroboides*, and *Populus deltoids*, which are in different stages of development. The experimental stand is a 14-year-old ginkgo timber plantation that lost seed source information.

**Fig. 2.**
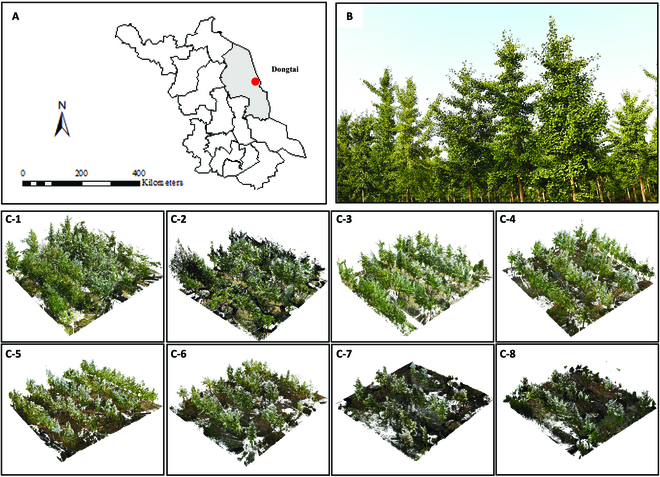
Study area description. (A) Geographical location. (B) Image of study site. (C-1 to C-8) Point cloud of plots1 to 8.

As shown in Fig. 2, a total of 8 plots (30 m × 30 m) were established and each plot was scanned by 7 to 8 scans on a clear, windless day in August 2020. Scanning was performed by RIEGL VZ-400i at standard geodetic sites identified in the plots, using multi-site scanning, with a pulse repetition frequency of 1,200 kHz and a range of up to 800 m. The vertical and horizontal scanning ranges were +60/−40 degrees and 0 to 360 degrees, respectively. Then, we registered the point cloud data of each site to integrate the 3D information of 8 plots. Out of 185 individual trees in the 8 plots, 102 individual trees were manually selected based on the criteria of being healthy, free of pests or diseases, and structurally well grown. The field measurement included DBH and H. Meanwhile, the fresh leaves were sealed in plastic bags, labeled, stored in containers with ice (0 °C), and transported to a −80 °C refrigerator for genetic experiments.

### DNA extraction, primer screening, and PCR amplification

Genomic DNA was extracted using the Magen HiPure SF Plant DNA Mini Kit following the manufacturer’s protocol. DNA solution (1 μl) was taken to determine the purity and concentration using Spectrophotometers (NanoDrop 2000, USA) and was normalized to 30 ng/μl for polymerase chain reaction (PCR).

The selected primers were derived from 2 parts: 13 pairs used in ginkgo-related studies in the published literature [17] and 8 pairs developed by our laboratory, for a total of 21 pairs of primers for the screening of sample-suitable SSR primers. PCR amplification was conducted using PrimeSTAR Max DNA Polymerase (R045B, TaKaRa, China). The reaction mixtures of 20 μl contained 10 μl of 2× Taq Master Mix (Nanjing Novozymes Biotechnology Co. Ltd.), 7 μl of ddH_2_O, 1 μl each of SSR forward and reverse primers, and 1 μl of genomic DNA template. The PCR program involved predenaturation at 95 °C for 3 min, denaturation at 95 °C for 10 s, appropriate annealing temperature for 10 s, extension at 72 °C for 30 s, 34 cycles, extension at 72 °C for 5 min, and storage at 4 °C.

### Genetic diversity and population structure analysis

The bands were assigned and counted, and those with equal distance and bright and clear bands on the electrophoresis map were counted as 1, while those without bands or with faint bands were counted as 0, forming a matrix of 1 and 0. The number of observed alleles (Na), the number of efficient alleles (Ne), the observed heterozygosity (Ho), the expected heterozygosity (He), adherence to Hardy–Weinberg equilibrium (assessed via a chi-square test), and Shannon's information index (I) were calculated by GenAlEx 6.5 [31]. Polymorphism information content index (PIC) was determined using Cervus 3.0 [32]. Neighbor-joining (NJ) dendrograms were obtained using R language based on Nei's genetic distance, and then results were imported into iTOL website for further optimization [33].

To estimate individual tree ancestry, we used STRUCTURE 2.3.4 to perform Bayesian clustering analysis on the sample set [34]. The genetic ancestry of each individual tree was estimated based on the admixture model. The *K* values were set to 1 to 20. Estimates were acquired using Markov chain Monte Carlo (MCMC) method with 500,000 iterations followed by a burn-in period of 100,000 iterations. The iterations were run 10 times independently. The resulting data were submitted to STRUCTURE HARVESTER [35], and the optimal *K* value was determined based on the distribution of Δ*K* [34]. Repeated sampling analysis was conducted via CLUMPP 1.1.2 [36], and DISTRUCT 1.1 [37] was used to plot the population structure.

### TLS data preprocessing and individual tree segmentation

To denoise the original point cloud data, we set a height threshold and classified the ground point clouds [37] using an improved progressive triangulated irregular network (TIN) densification filtering algorithm [38], generating the digital elevation model (DEM). By subtracting the elevation value of each point from the DEM elevation value, the CHM was generated to obtain the vertical information of the trees excluding the influence of the ground. The segmentation algorithm was the CSP algorithm for terrestrial LiDAR and mobile LiDAR [39], which included 2 steps, trunk detection and crown segmentation, with the latter inspired by the proven theory of metabolic ecology and the ecological fact that vascular plants prefer to minimize the transfer distance to the roots.

### Extraction of growth structural characteristics

**Fig. 3.**
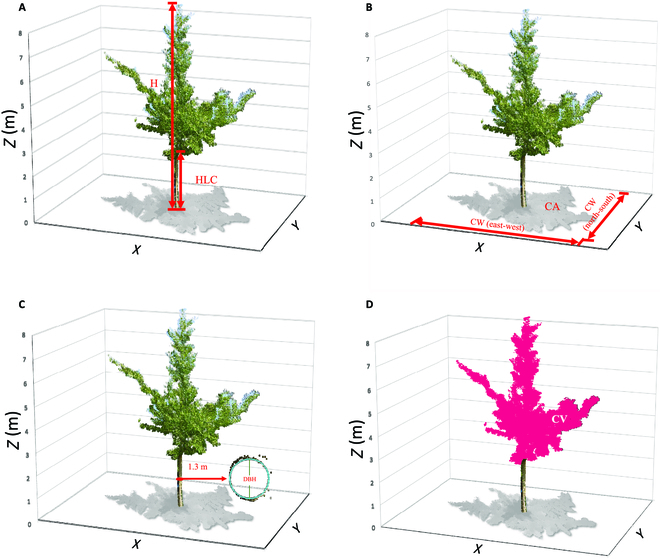
Measurement of H and HLC (A), CA and north–south and east–west CW (B), DBH (C), and CV (D) of example individual tree (G318).

After individual tree segmentation, the individual tree characteristics such as H, CA, and CV were extracted. H is the vertical height from the ground to the treetop. HLC, which is the vertical height of the first living branch from the ground surface to the tree crown, was calculated according to the vertical distribution of tree point cloud. The CW in the north–south and east–west directions was extracted from the point cloud projection and averaged as the final value. CA was obtained by calculating the crown projection area, and CV was obtained by calculating the volume of fill, cut, and cut-and-fill. Since some trunk point clouds were incomplete or obscured by branches or leaves at breast height, the point clouds fitted automatically did not match the actual point clouds, so the sliced point clouds at 1.3 m were selected from the detected trunks, and DBH was obtained by DBSCAN algorithm (Fig. 3).

Biomass of all components was calculated using the ginkgo plantation allometric model developed by Liu et al. [40]. The subject of Liu’s study is also a ginkgo plantation and is geographically similar to ours. Thirteen ginkgo individuals were selected for whole-plant excavation, and the biomass models were established with DBH, H, DBH^2^H, and DBH^a^H^b^ as independent variables. The optimal fitting models at all levels were identified based on the test results of parameter estimation. The optimal models are shown in Table 1.

**Table 1.** Optimal allometric model of biomass of all components.

Biomass	Optimal allometric model
Leaf biomass (LB)	ln*W* = − 5.21 + 2.37 ln *DBH*
Branch biomass (BB)	ln*W* = − 6.95 + 3.36 ln *DBH*
Trunk biomass (TB)	ln*W* = − 1.61 + 2.27 ln* DBH* − 0.36 ln* H*
Above-ground biomass (AGB)	ln*W* = − 2.56 + 2.40 ln *DBH*
Root biomass (RB)	ln*W* = − 2.64 + 2.63 ln *DBH* − 0.64 ln *H*
Whole-plant biomass (WPB)	ln*W* = − 1.01 + 2.61 ln *DBH* − 0.73 ln *H*

To obtain the TV, we reconstructed the 3D branch geometry of individual trees using QSM based on high-density point clouds [41]. The method first reconstructed the topology of the whole tree by segmenting TLS point clouds (0.5 m) and then reconstructed the surface and volume of the segment by fitting cylinders to all segments to calculate the volume of the woody part of individual trees.

### Evaluation of data and comprehensive evaluation

The accuracy of H was evaluated on the basis of field-measured data by adjusted coefficient of determination (Adj-*R*^2^), root mean square error (RMSE), and relative root mean square error (rRMSE) [42], and the measured values were compared with the extracted values, which were calculated as follows:

$$\begin{array}{c} \text{Adj}-R^{2}=1-\frac{\sum_{i=1}^{n}{\left(x_{i}-{\hat{x}}_{i}\right)}^{2}}{\sum_{i=1}^{n}{\left(x_{i}-{\bar{x}}_{i}\right)}^{2}}\\ \text{RMSE}=\sqrt{\frac{1}{n}\sum_{i=1}^{n}(x_{i}-\hat{x_{i}}{)}^{2}}\\ \text{rRMSE}=\frac{\text{RMSE}}{\bar{x}}\times 100\% \end{array}$$

where *x_i_* is the value of a characteristic, $\bar{x_{i}}$ is the average value of a characteristic, $\hat{x_{i}}$ is the model estimated value of a characteristic, and *n* is the number of individual trees.

Processed by R language, correlation analysis was performed using Spearman's method. PCA can remove redundant information through projection and dimensionality reduction without any artificial weighting, and assigns corresponding scores to each characteristic of individual trees, so it is commonly used as a comprehensive evaluation method. Growth structural characteristics were evaluated via PCA using SPSS 25.0 within each genetic group. Due to the far genetic distance among groups, we ensured that the screened trees were genetically distant from each other among groups, which allowed the screened superior trees to retain a certain amount of genetic diversity. Trees with the highest comprehensive ratings were selected as the superior tree (the inclusion rate set at 10%).

## Results

### Analysis of genetic diversity

Fourteen pairs of SSR primers (Table S1) with good repeatability, high stability, and clear amplification bands are selected from 21 pairs of primers (Fig. S1), with annealing temperatures of 51 to 57 °C and marker fragment lengths of 117 to 295 base pairs.

The polymorphism analysis of 14 SSR primers (Table S2) shows that a total of 114 alleles are obtained based on 102 individual trees, with an average of 8.14 alleles per locus varying from 3 to 12. Ne ranges from 1.622 to 7.437, with an average of 4.76 alleles. PIC ranges from 0.363 to 0.850, with an average of 0.719, indicating a high level of genetic diversity among 102 individual trees. Ho is 0.477, He is 0.749, and I is 1.672. The highest number of alleles is detected at locus C394, with the largest He and I (0.858 and 2.155). The E-SSR202 locus has the largest Ne (7.437), and the G-SSR279 locus has the largest Ho (1.000). Moreover, except for E-SSR354 and G-SSR279, Ho of all 12 loci is lower than He, implying the presence of heterozygosity deficiency in this ginkgo plantation, which is generally an indication of inbreeding [43]. However, allele frequencies of 13 SSR loci deviate from Hardy–Weinberg equilibrium markedly (*P* < 0.01). The high number of valid alleles for E-SSR202, C394, and C388 indicates their higher importance in the population structure of the population, while E-SSR32 and E-SSR354 have fewer effective alleles and play a less important role. In general, the 14 primers selected have high genetic diversity and could meet the corresponding analysis requirements.

### Analysis of population structure

**Fig. 4.**
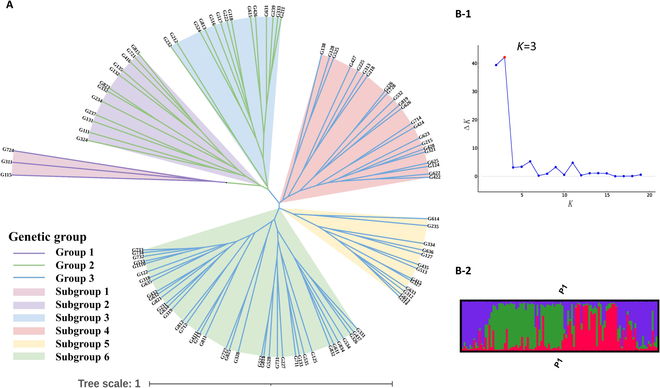
(A) Phylogenetic tree of 102 ginkgo individuals. (B) Results of population structure analyzed by program Structure. (B-1) Magnitude of Δ*K* (*k* = 3). (B-2) Histogram of individual assignments.

Neighbor-joining clustering is used to obtain the relationship of Nei's genetic distance of 102 ginkgo individuals. The results (Fig. 4) show that the 102 individual trees fell into 3 groups and could be further divided into 6 subgroups, with individual trees G311, G724, and G115 clustered into one group (group 1). It can be seen that G311, G724, and G115 are more distantly related to the remaining individual trees.

Analyzing co-ancestry relations of the plantation population is based on the Bayesian clustering model. The optimal population number *K* is determined for 102 individual trees according to the Δ*K* result. The optimal *K* value is 3 when Δ*K* reaches the peak (Fig. 4B-1), and all individual trees can be classified into 3 groups, which is basically consistent with the NJ dendrogram. Inflection points are observed when *K* = 6, 9, or 11. Figure 4B-2 shows the population structure diagram of the ginkgo plantation population, each vertical line represents an individual tree, and the color of its composition represents the ancestral populations to which the individual tree belongs. The population structure diagram shows that no individual tree is 100% from one ancestral population, and there are large genetic differences between different individual trees.

### Individual tree segmentation

**Fig. 5.**
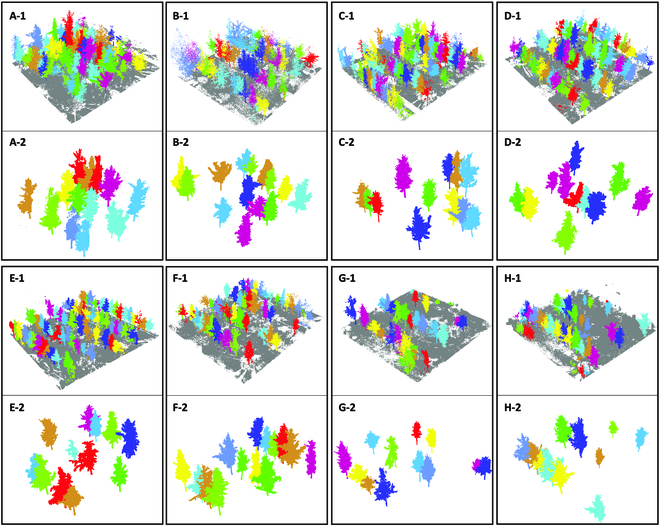
Individual tree segmentation results with the TLS data. (A-1 to H-1) Segmentation results of plots 1 to 8. (A-2 to H-2) Segmentation results of selected individual trees in plots 1 to 8.

As shown in Fig. 5, point clouds of different colors represent different individual trees, and gray point clouds represent the ground. The tree information of the TLS data is relatively comprehensive, while a few point clouds of the ground data are concluded into point clouds of trees. The missing or redundant point clouds are adjusted to obtain more accurate individual tree segmentation.

### Growth structural characteristic analysis

The growth structural characteristics of the 102 individual trees are summarized in Table 2. There are 5 statistical indicators, including mean, standard deviation (SD), coefficient of variation (CV), maximum (Max), and minimum (Min). CV of the 13 growth structural characteristics ranges from 21.822% to 85.477%, and the CV of crown area, crown volume, trunk volume, and biomass of all components are more than 50%, indicating that the dispersion degree is large for each growth structural characteristic, especially for crown area, crown volume, trunk volume, and biomass of all components.

**Table 2.** Statistics of the measured growth structural characteristics.

No	Characteristic	Abbreviation	Mean	SD	CV (%)	Max	Min
*X*_1_	Height	H	7.757	1.693	21.822	12.057	4.132
*X*_2_	Diameter at breast height	DBH	13.233	3.394	25.645	22.700	5.900
*X*_3_	Crown width	CW	4.254	1.112	26.132	7.617	2.165
*X*_4_	Crown area	CA	10.073	5.467	54.274	29.525	2.187
*X*_5_	Crown volume	CV	32.321	19.573	60.559	96.153	4.760
*X*_6_	Trunk volume	TV	0.089	0.048	54.414	0.259	0.010
*X*_7_	Height to living crown	HLC	2.094	0.502	23.977	3.358	0.885
*X*_8_	Leaf biomass	LB	2.819	1.679	59.546	9.159	0.376
*X*_9_	Branch biomass	BB	7.680	6.565	85.477	37.163	0.402
*X*_10_	Trunk biomass	TB	35.899	18.147	50.552	97.680	6.003
*X*_11_	Above-ground biomass	AGB	42.223	25.463	60.305	138.931	5.475
*X*_12_	Root biomass	RB	18.632	10.186	54.668	53.471	2.494
*X*_13_	Whole-plant biomass	WPB	74.507	39.308	52.758	204.820	10.499

**Fig. 6.**
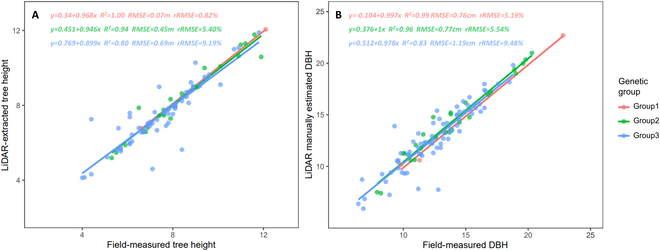
Accuracy assessment of H and DBH extraction based on TLS data. (A) H. (B) DBH.

**Fig. 7.**
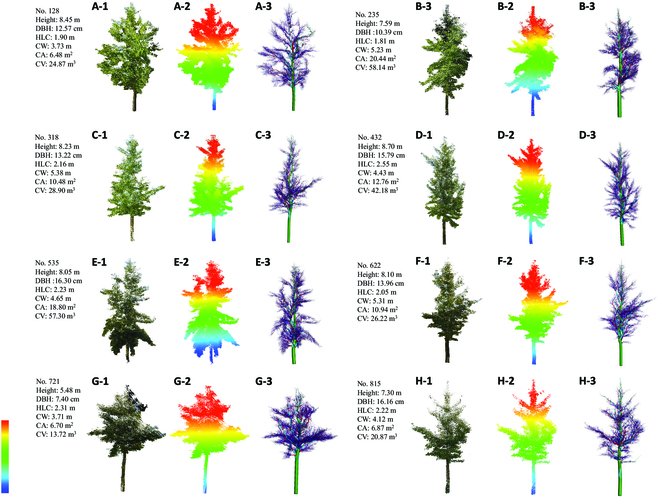
Reconstruction result of example individual tree of plots 1 to 8. (A-1 to H-1) Point cloud. (A-2 to H-2) Displayed by height. (A-3 to H-3) QSM visualization.

For the 102 samples of 3 genetic groups, the field-measured H and DBH values, and LiDAR-estimated H and DBH values have a good consistency. The Adj-*R*^2^, RMSE, and rRMSE range of field-measured and LiDAR-estimated H is 0.80 to 1.00, 0.07 to 0.69 m, and 0.82% to 9.19%, respectively (Fig. 6A), and the Adj-*R*^2^, RMSE, and rRMSE range of field-measured and LiDAR-estimated DBH is 0.83 to 0.99, 0.76 to 1.19 cm, and 5.19% to 9.48% (Fig. 6B). The good consistency indicates that the individual tree H and DBH derived from TLS data have high estimation accuracy. From the case of the 3D model, the QSM fits the tree morphology well. Example point clouds and QSM of individual trees are shown in Fig. 7.

**Fig. 8.**
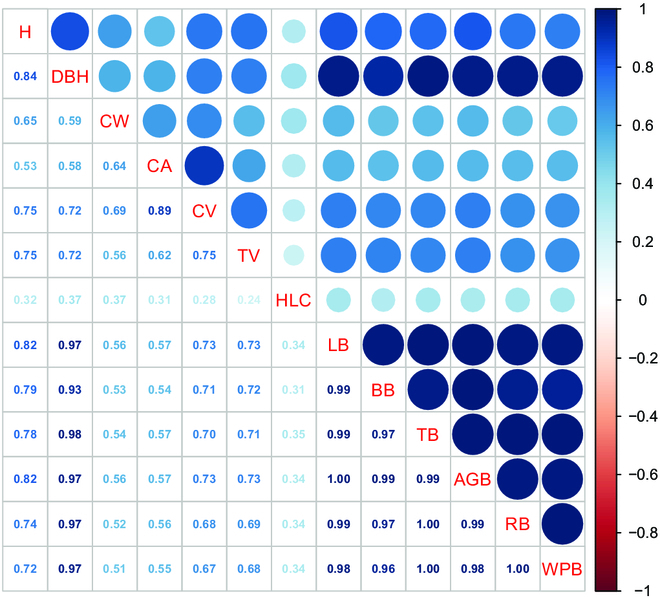
Correlation coefficient between growth structural characteristics.

The results of the correlation analysis of 13 growth structural characteristics are shown in Fig. 8. All growth structural characteristics are positively correlated. There is a positive correlation between the biomass of all components, which indicates that if the biomass of one component is high, the biomass of other components will also be high. DBH and biomass of all components are strongly positively correlated, indicating that DBH has great potential in estimating biomass. HLC has little correlation with all other growth structural characteristics. There is a strong positive correlation between CA and CV, but CW does not have a strong positive correlation with CA and CV.

### PCA and comprehensive evaluation

PCA is performed on the 13 growth structural characteristics of individual trees in 3 genetic groups, and the principal component (PC) load matrix and variance contribution rates are shown in Tables 3 to 5. Based on the criteria of cumulative contribution rate (CCR) of 85%, 1 PC (group 1), 2 PCs (group 2), and 3 PCs (group 3) are extracted. The CCR of the PCs reached 95.8% (group 1), 87.445% (group 2), and 85.631% (group 3), respectively, which meant that the selected PCs represent most of the comprehensive information of the 13 characteristics. Therefore, the selected PCs can reflect the relative importance of the 13 characteristics and the relationship between the characteristics. In order to reduce dimension, these PCs are used as comprehensive rating indices.

**Table 3.** Principal component load matrix and variance contribution rate of the growth structural characteristics of *G. biloba* in genetic group 1.

	Principal component loading
	PC 1
Height	0.996
Diameter at breast height	0.996
Crown width	−0.829
Crown area	0.985
Crown volume	1.000
Trunk volume	0.989
Height to living crown	0.950
Leaf biomass	0.994
Branch biomass	0.994
Trunk biomass	0.995
Above-ground biomass	0.994
Root biomass	0.995
Whole-plant biomass	0.995
Eigenvalues	12.454
Contribution rate/%	95.800
Cumulative contribution rate/%	95.800

**Table 4.** Principal component load matrix and variance contribution rate of the growth structural characteristics of *G. biloba* in genetic group 2.

	Principal component loading	
	PC 1	PC 2
Height	0.936	−0.113
Diameter at breast height	0.974	0.108
Crown width	0.866	−0.092
Crown area	0.785	−0.348
Crown volume	0.841	−0.370
Trunk volume	0.794	−0.417
Height to living crown	0.241	0.707
Leaf biomass	0.985	0.113
Branch biomass	0.976	0.111
Trunk biomass	0.975	0.150
Above-ground biomass	0.985	0.113
Root biomass	0.964	0.175
Whole-plant biomass	0.958	0.187
Eigenvalues	10.277	1.091
Contribution rate/%	79.051	8.394
Cumulative contribution rate/%	79.051	87.445

**Table 5.** Principal component load matrix and variance contribution rate of the growth structural characteristics of *G. biloba* in genetic group 3.

	Principal component loading		
	PC 1	PC 2	PC 3
Height	0.779	0.088	−0.143
Diameter at breast height	0.979	−0.119	0.040
Crown width	0.141	0.417	0.863
Crown area	0.596	0.662	−0.213
Crown volume	0.769	0.504	−0.274
Trunk volume	0.734	0.289	−0.147
Height to living crown	0.422	0.273	0.162
Leaf biomass	0.982	−0.175	0.035
Branch biomass	0.964	−0.208	0.036
Trunk biomass	0.976	−0.185	0.064
Above-ground biomass	0.982	−0.176	0.035
Root biomass	0.963	−0.204	0.081
Whole-plant biomass	0.957	−0.206	0.088
Eigenvalues	8.906	1.270	0.956
Contribution rate/%	68.504	9.771	7.356
Cumulative contribution rate/%	68.504	78.275	85.631

These PCs reveal the comprehensive conditions of ginkgo individual tree growth. With *X_1_, X_2_, X_3_ ……X_13_* representing the standardized values of the original data of the indicators, and *F_n_* representing the score of the *n*th PC, the linear equation of the scores of each PC is obtained according to the eigenvector matrix (Table S3). The scores of each PC are calculated separately according to the above linear equation, and the contribution of each PC is used as the weight to calculate the combined score of each genetic group. Comprehensive score calculation formulas are as follows.

$$\text{Group}\;1:F=95.8\%F_{1}$$

$$\text{Group}\;2:F=79.051\%F_{1}+8.394\%F_{2}$$

$$\text{Group}\;3:F=68.504\%F_{1}+9.771\%F_{2}+7.356\%F_{3}$$

Data of the 102 individual trees of different genetic groups are standardized, and their scores on the selected PCs are obtained (Tables S4 to S6). According to comprehensive scores, the individual trees are ranked and superior individual trees are screened out. The inclusion rate is set to 10%, and although there are only 3 individual trees in group 1, they are distantly related to individual trees in other groups, so one individual tree is still needed to be screened out in group 1. The overall scores of the selected individual trees are shown in Tables 6 to 8. The selected individual trees are G115 (group 1); G135, G132, and G111 (group 2); and G334, G122, G138, G415, G231, G534, and G1110 (group 3).

**Table 6.** Comprehensive evaluation in growth structural characteristics of selected individuals of genetic group 1.

Individual no.	*F*_1_	Overall scores	Rank
G115	653.207	625.772	1

**Table 7.** Comprehensive evaluation in growth structural characteristics of selected individuals of genetic group 2.

Individual no.	*F*_1_	*F*_2_	Overall scores	Rank
G135	569.482	33.754	453.014	1
G132	524.084	32.352	417.009	2
G111	486.499	42.763	388.172	3

**Table 8.** Comprehensive evaluation in growth structural characteristics of selected individuals of genetic group 3.

Individual no.	*F*_1_	*F*_2_	*F*_3_	Overall scores	Rank
G334	478.100	−59.841	21.507	323.253	1
G122	392.492	−14.432	−0.074	267.457	2
G138	387.638	−23.626	5.440	263.639	3
G415	385.821	−55.052	20.133	260.405	4
G231	377.654	−24.750	7.718	256.858	5
G534	373.020	−6.028	1.590	255.062	6
G1110	371.150	−25.586	8.358	252.367	7

## Discussion

In this study, we applied TLS high-throughput phenotype acquisition technology for the selection and breeding of Ginkgo plantations, which is seldom seen in the previous literature. We used TLS data to characterize the growth of individual trees in a ginkgo plantation and divided individual trees into 3 genetic groups according to the genetic distance. The superior individual trees with the best growth of each genetic group were selected using PCA. Our study enables the screened individual trees to retain the genetic information of each group as much as possible. Combined with phenotype screening, the screened population of superior trees can preserve both phenotypic advantages and genetic diversity. The results reveal the potential and feasibility of ground-based LiDAR in acquiring and analyzing high-throughput phenotypic characteristics of individual trees, as well as the prospect of combining remote sensing technology with genetic experiments for analysis.

### Abundant genetic variation within ginkgo populations

Many studies showed that the ginkgo population has high genetic diversity [17,44,45], and this is also the case in our study. Fourteen pairs of SSR primers with good polymorphism were selected to amplify 102 ginkgo plantation individuals with clear bands, Ho of 0.477, He of 0.749, I of 1.672, and PIC of 0.719, indicating that the ginkgo plantation possesses high genetic diversity. One hypothesis is that the seed source might be high-quality genetic resources, so the plantation inherited high genetic diversity. Another hypothesis is that ginkgo is dioecious, and pollen is spread by wind and can travel long distances, which allows strong genetic exchange between populations [46,47]. Climate change in different areas and frequent introgression and hybridization between regions are conducive to the accumulation and maintenance of genetic variation, allowing ginkgo to exhibit rich genetic diversity.

The complex genetic background may be caused by the evolutionary history of ginkgo. The population structure of plant populations is influenced by various factors such as life history, geographic distribution, and mating system [17,48]. The study of population structure can measure the richness of population variation and adaptability to the environment. Ginkgo is a relict plant from the surviving Quaternary glaciers, and its ancestors have a relatively rich genetic base [49]. In a previous study on the population structure of male ginkgo populations, Zhou et al. [17] found a low degree of genetic differentiation among 7 ginkgo populations, with only 9.4% of the variation existing among populations and 90.6% of the genetic variation existing within populations. Similarly, Tang’s study indicates that genetic variations were primarily from variations within the families [50]. In this study, the population structure of 102 ginkgo plantation individual trees was analyzed by structure and phylogenetic analysis, and the results of structure and phylogenetic analysis were consistent. One hundred two individual trees could be divided into 3 populations, and individual trees G311, G724, and G115 showed more distant affinities with other samples. It was speculated that the seeds planted in this plantation might come from 3 populations. The population structure diagram can visually show the genetic components of each individual tree and the proportion of each component [37], and it can be seen that no individual tree is 100% from one population, suggesting that there is great genetic variation among the individual trees within this ginkgo plantation and that the genetic background is complex, which may be the result of the more complex population structure and higher heterozygosity of ginkgo through long-term adaptation and evolution. In general, the higher the genetic diversity of the plantation, the richer the allelic variation, and the higher the genetic diversity of the population formed by selection. Therefore, the plantation under this study can be used as a suitable selection population.

### Availability of TLS point cloud data in quantifying individual tree growth

TLS has been identified as an efficient technique for extracting highly detailed and accurate structural information of individual trees. Currently, it can provide 3D point cloud data with the best detail and accuracy for extracting forest-related variables [51]. Since TLS has better access to trunk information, it is more advantageous in analyzing individual tree information [52]. However, the acquisition of TLS 3D point clouds is susceptible to weather, mainly wind. Even very light winds can lead to the misestimation of tree morphological indices, especially for the top of the crown [53]. In this study, the individual tree segmentation results of using the algorithm specially developed for terrestrial LiDAR and mobile LiDAR are quite satisfactory, while manual adjustment is still necessary for erroneous point clouds. Generally, only individual trees with intervals higher than or equal to 4 m can be automatically divided with accuracy [23]. It is rather processing to adjust the individual tree segmentation result manually. Especially, for overlapping parts of the crown, even manual segmentation may fail to achieve excellent results.

Tree growth structural characteristics can be extracted using TLS. The results of scatterplots indicated that, based on the accurate segmentation result, there was a high correlation between H and DBH within different genetic groups, indicating that LiDAR-extracted H and DBH could reveal the real situation well. For the calculation of biomass, there are direct measurement methods and indirect estimation methods. The former is more accurate but costly and highly destructive to local resources [54]; the latter, such as relative growth models, is now frequently used [55]. Since the allometric models used in this study were developed based on ginkgo plantations with similar environments and cultivation [40], they are well suited for biomass calculation in this study. From the correlation analysis, it was found that the biomass of each component was significantly and positively correlated with DBH and less positively correlated with H. Notably, when developing a model, the introduction of another parameter may reduce the accuracy of the description if one parameter can already describe the correlation well [56]. Moreover, there is a need to develop more models for different species and different cultivation conditions in the calculation of biomass. Today’s well-established models are not sufficient for the investigation of individual tree characteristics of common tree species. In this study, QSM fitted the tree morphology well, and also, accurate wood volume estimates using QSM for different tree species in different forest systems have been reported, such as urban forests [57], subtropical planted forests [58], crop-livestock forests [59], and tropical forests [60].

There is quite a bit of variation in the growth structural characteristics of individual trees in this plantation, according to the statistical analysis. The dispersion degree is large for each growth structural characteristic, which may be caused by the high genetic diversity of the population. However, since most growth structural characteristics, such as height [61], biomass [62], and DBH [61], have low heritabilities, they are also easily influenced by the environment [63]. Although the silvicultural measures of the individual trees are basically the same, it is hard to eliminate all possible interferences of the environment.

### Combining genetic analysis and remote sensing technologies for superior individual tree selection

Combining genetic analysis and remote sensing technologies is beneficial for the in-depth understanding of plantation resources, further providing a basis for the strategy of genetic resource conservation and utilization, and accelerates the screening and breeding of superior individual trees. In this study, genetic analysis and remote sensing technology were combined to accelerate forest breeding. We first divided the tree into different genetic groups according to the genetic distance, then quantified the growth structural characteristics using TLS, and finally used PCA to screen out trees with superior growth structural characteristics in each genetic group, thus preserving as much genetic diversity as possible while guaranteeing the best growth structural characteristics. It is likely that these ideas could be applied to other tree species planted under similar frameworks in the future, especially for large-scale plantations where no information on seed sources is available. With the development of computer science, surveying technology, and geographic information technology, the integration of remote sensing with various disciplines is opening up new possibilities for forest resource management [64]. Using remote sensing and traditional forest inventory methodologies, forest precision monitoring can be performed with high accuracy and throughput [65]. In addition to superior tree selection and breeding, the intersection of genetics and remote sensing technologies can solve a range of forestry problems. Limited by traditional phenotype acquisition methods, it is difficult to obtain sufficient phenotypes for genetic association analysis in quantitative trait function studies [21]. High-throughput phenotype monitoring makes it possible to obtain a large number of phenotypes to assist in functional studies of quantitative traits [66]. Remote sensing techniques can also identify and quantify biotic and antibiotic injuries at high throughput, such as fungal disease [67], frost damage [68], and different kinds of stress [69], combined with population genetics, leading to predictions of vulnerable populations. The integration of remote sensing technology with various disciplines holds great prospects and is the key to solving a range of forestry problems.

## Conclusion

In this study, we displayed the ability of high-density TLS to characterize the growth of ginkgo plantation individual trees in a breeding process. Combined with genetic analysis, 102 individual trees were comprehensively assessed using PCA and 11 superior individual trees were screened out for future breeding. The rational use of plantation resources is an essential issue that deserves in-depth consideration by researchers and forest resource management departments. Remote sensing provides an effective way to phenotype forests due to its fast operation and easy deployment. The results of the study showed the high genetic diversity and complex population structure within the ginkgo plantation. Meanwhile, the accuracy of TLS in extracting individual tree structural characteristic information was demonstrated. In addition, the combined utilization of genetic analysis and TLS enabled the screened trees to have as much phenotypic advantage and genetic diversity as possible, which provides insights into the application of remote sensing technologies to accelerate the forest breeding process.

## Data Availability

The data used in this study are freely available. Anyone who wants to use the data can contact the corresponding author X.S. The author is with the Co-Innovation Center for Sustainable Forestry in Southern China, Nanjing Forestry University, Nanjing 210037, China (email: xinshen@njfu.edu.cn).
