# Supplementary material for: Screening of Ginkgo Individuals with Superior Growth Structural Characteristics in Different Genetic Groups Using Terrestrial Laser Scanning (TLS) Data
Source: Plant Phenomics. 2023 Sep 22;5:0092. doi: 10.34133/plantphenomics.0092 (PMC10515975; doi:10.34133/plantphenomics.0092)
Supplement: Supplementary 1 — Fig. S1 Tables S1 to S6 [file plantphenomics.0092.f1.docx]

Supplementary Materials


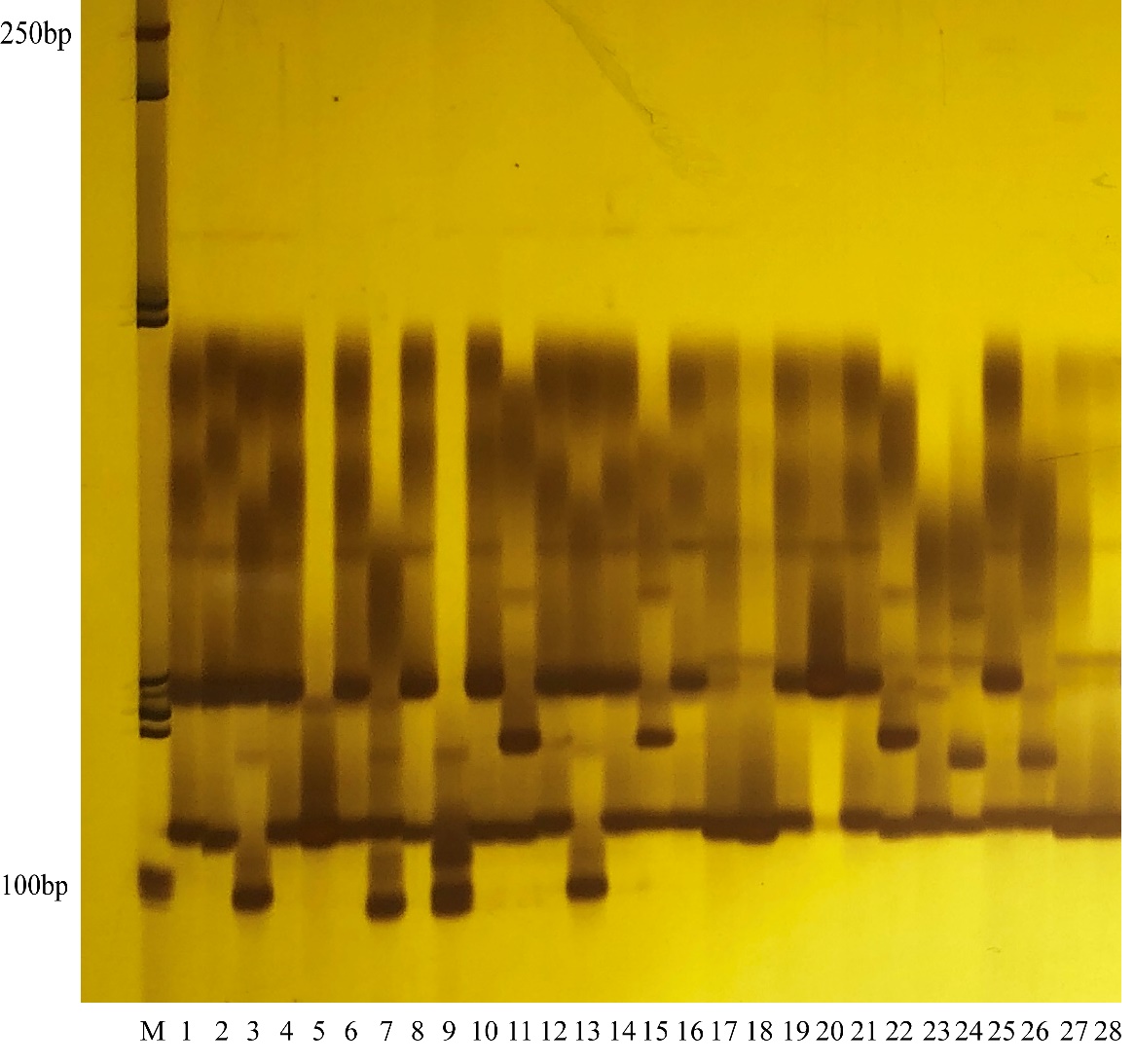


F_IGURE_ S1: Amplification of primer C412 in some samples

T_ABLE_ S1: Repeat motif, primer sequence, Tm, fragment size and information for14 SSR loci.

| Locus  code | Repeat  motif | Primer Sequence | Tm (℃) | Fragment size  (bp) |
| --- | --- | --- | --- | --- |
| E-SSR32 | (TA)11 | F: TTCGCTGTAGCATTTGTG | 57 | 196 |
|  |  | R: GCAGGTTGTATTTCGGAG |  |  |
| E-SSR120 | (CA)11 | F: AAGTCATAAGCGACAGTG | 55 | 234 |
|  |  | R: CCGTCTTTCAGATCAATA |  |  |
| E-SSR202 | (AG)14 | F: CCCTTGTTTCTCCATAAT | 55 | 267 |
|  |  | R: TGCTCATATAGGTGCTCT |  |  |
| E-SSR354 | (TG)11 | F: GATGAAGTGTGAAGAGAATG | 55 | 295 |
|  |  | R: AACTACGATGACGATGGA |  |  |
| G-SSR258 | (TA)11 | F: GACTTTTGGCACTATCGT | 57 | 225 |
|  |  | R: GTCAATGGGAGACAGGTT |  |  |
| G-SSR279 | (TG)12 | F: TTGATTTTTCTCTCCGTC | 55 | 202 |
|  |  | R: CCTTTAGCACATTTCCA |  |  |
| G-SSR448 | (TC)10 | F: GTTCAAGGTCCTCATAG | 55 | 156 |
|  |  | R: TAGCCTCTTCTTTACTG |  |  |
| C110 | (AT)12 | F: GGAGAGTCATGTTGTTATAAGAGGGA | 55 | 199 |
|  |  | R: TGTTGATGAACCTACGACAAGAGT |  |  |
| C114 | (TATC)7 | F: ACCTTATGGAAATTATTTGCCATGGA | 54 | 178 |
|  |  | R: CATTTTATTGTGCATGTAACAAACACA |  |  |
| C217 | (AT)17 | F: AGGACTCTATAGAGGTCTAATTCCCT | 55 | 139 |
|  |  | R: AGACCTCTATGCAGTCTCTTGA |  |  |
| C394 | (TATC)6 | F: TGTTCCCACTTACCATTATGAGAT | 53 | 191 |
|  |  | R: ACTTTGTTGATTACTTCACTAGCCT |  |  |
| C412 | (TCTTTA)4 | F: ACATGCGAAAACATTCCATTCCA | 55 | 117 |
|  |  | R: CCAATGAGGCAACGTTGGTT |  |  |
| C388 | (TA)12 | F: CATCTTTGCAACGTAATTCAACTAAA | 51 | 187 |
|  |  | R: AGATTTCTGTCTACCTCTGCAAT |  |  |
| C958 | (AT)16 | F: ACTAGTAGGGTTTTGGGTGATTT | 53 | 199 |
|  |  | R: TCGTTCATCTACCTACTAAGTAACAAC |  |  |

T_ABLE_ S2: Polymorphism analysis of SSR primers

| Primer Name | *Na* | *Ne* | *Ho* | *He* | *I* | *PIC* | *HWE* |
| --- | --- | --- | --- | --- | --- | --- | --- |
| E-SSR32 | 6 | 1.622 | 0.333 | 0.383 | 0.822 | 0.363 | ns |
| E-SSR120 | 8 | 4.450 | 0.402 | 0.775 | 1.734 | 0.751 | *** |
| E-SSR202 | 9 | 7.437 | 0.647 | 0.866 | 2.068 | 0.850 | *** |
| E-SSR354 | 3 | 2.220 | 0.990 | 0.550 | 0.866 | 0.447 | *** |
| G-SSR258 | 5 | 3.995 | 0.088 | 0.750 | 1.469 | 0.707 | *** |
| G-SSR279 | 8 | 5.716 | 1.000 | 0.825 | 1.898 | 0.804 | *** |
| G-SSR448 | 8 | 6.162 | 0.216 | 0.838 | 1.940 | 0.818 | *** |
| C110 | 7 | 3.723 | 0.245 | 0.731 | 1.537 | 0.691 | *** |
| C114 | 9 | 4.943 | 0.559 | 0.798 | 1.795 | 0.772 | *** |
| C217 | 11 | 4.901 | 0.235 | 0.796 | 1.882 | 0.773 | *** |
| C394 | 12 | 7.027 | 0.696 | 0.858 | 2.155 | 0.843 | *** |
| C412 | 11 | 4.074 | 0.608 | 0.755 | 1.696 | 0.720 | *** |
| C388 | 9 | 6.793 | 0.245 | 0.853 | 2.001 | 0.835 | *** |
| C958 | 8 | 3.523 | 0.412 | 0.716 | 1.541 | 0.684 | *** |
| Mean | 8.14 | 4.756 | 0.477 | 0.749 | 1.672 | 0.719 | - |

T_ABLE_ S3: Linear equation of each principal component score

| Genetic group | *F_n_* |
| --- | --- |
| Group 1 | $F_{1}=0.996X_{1}+0.996X_{2}-0.829X_{3}+0.985X_{4}+X_{5}+0.989X_{6}+0.95X_{7}+0.994X_{8}-0.994X_{9}+0.995X_{10}+0.994X_{11}+0.995X_{12}+0.995X_{13}$ |
| Group 2 | $F_{1}=0.936X_{1}+0.974X_{2}+0.866X_{3}+0.785X_{4}+{0.841X}_{5}+0.794X_{6}+0.241X_{7}+0.985X_{8}+0.976X_{9}+0.975X_{10}+0.985X_{11}+0.964X_{12}+0.958X_{13}$ |
|  | $F_{2}=-0.113X_{1}+0.108X_{2}-0.092X_{3}-0.348X_{4}-0.37X_{5}-0.417X_{6}+0.707X_{7}+0.113X_{8}+0.111X_{9}+0.15X_{10}+0.113X_{11}+0.175X_{12}+0.187X_{13}$ |
| Group 3 | $F_{1}=0.779X_{1}+0.979X_{2}+0.141X_{3}+0.596X_{4}+0.769X_{5}+0.734X_{6}+0.422X_{7}+0.982X_{8}+0.964X_{9}+0.976X_{10}+0.982X_{11}+0.963X_{12}+0.957X_{13}$ |
|  | $F_{2}=0.088X_{1}-0.119X_{2}+0.417X_{3}+0.662X_{4}+0.504X_{5}+0.289X_{6}+0.273X_{7}-0.175X_{8}-0.208X_{9}-0.185X_{10}-0.176X_{11}-0.204X_{12}-0.206X_{13}$ |
|  | $F_{3}=-0.143X_{1}+0.04X_{2}+0.863X_{3}-0.213X_{4}-0.274X_{5}-0.147X_{6}+0.162X_{7}+0.035X_{8}+0.036X_{9}+0.064X_{10}+0.035X_{11}+0.081X_{12}+0.088X_{13}$ |

T_ABLE_ S4: Comprehensive evaluation in growth structural characteristics among *G. biloba* of genetic group 1

| Individual No. | *F1* | Overall ratings | Rank |
| --- | --- | --- | --- |
| G115 | 653.207 | 625.772 | 1 |
| G724 | 146.048 | 139.914 | 2 |
| G311 | 124.256 | 119.038 | 3 |

T_ABLE_ S5: Comprehensive evaluation in growth structural characteristics among *G. biloba* of genetic group 2

| Individual No. | *F1* | *F2* | Overall ratings | Rank |
| --- | --- | --- | --- | --- |
| G135 | 569.482 | 33.754 | 453.014 | 1 |
| G132 | 524.084 | 32.352 | 417.009 | 2 |
| G111 | 486.499 | 42.763 | 388.172 | 3 |
| G118 | 478.055 | 17.975 | 379.416 | 4 |
| G131 | 471.779 | 23.057 | 374.881 | 5 |
| G239 | 413.917 | 2.389 | 327.406 | 6 |
| G211 | 351.844 | 23.120 | 280.077 | 7 |
| G815 | 324.279 | 36.851 | 259.439 | 8 |
| G237 | 301.649 | 17.479 | 239.924 | 9 |
| G333 | 272.079 | 23.584 | 217.061 | 10 |
| G813 | 269.021 | 32.781 | 215.415 | 11 |
| G212 | 264.810 | 16.984 | 210.761 | 12 |
| G234 | 266.313 | -10.599 | 209.633 | 13 |
| G615 | 249.787 | 18.776 | 199.035 | 14 |
| G222 | 240.624 | 8.118 | 190.897 | 15 |
| G416 | 230.946 | 7.921 | 183.230 | 16 |
| G426 | 208.166 | 17.308 | 166.010 | 17 |
| G516 | 203.796 | 11.943 | 162.106 | 18 |
| G332 | 178.534 | 10.998 | 142.056 | 19 |
| G823 | 171.216 | 6.997 | 135.936 | 20 |
| G517 | 168.729 | 10.126 | 134.232 | 21 |
| G232 | 158.928 | 4.684 | 126.028 | 22 |
| G631 | 148.077 | 5.721 | 117.537 | 23 |
| G324 | 140.047 | 6.552 | 111.259 | 24 |
| G721 | 76.835 | 1.279 | 60.846 | 25 |
| G524 | 70.524 | 5.212 | 56.187 | 26 |

T_ABLE_ S6: Comprehensive evaluation in growth structural characteristics among *G. biloba* of genetic group 3

| Individual No. | *F1* | *F2* | *F3* | Overall ratings | Rank |
| --- | --- | --- | --- | --- | --- |
| G334 | 478.100 | -59.841 | 21.507 | 323.253 | 1 |
| G122 | 392.492 | -14.432 | -0.074 | 267.457 | 2 |
| G138 | 387.638 | -23.626 | 5.440 | 263.639 | 3 |
| G415 | 385.821 | -55.052 | 20.133 | 260.405 | 4 |
| G231 | 377.654 | -24.750 | 7.718 | 256.858 | 5 |
| G534 | 373.020 | -6.028 | 1.590 | 255.062 | 6 |
| G1110 | 371.150 | -25.586 | 8.358 | 252.367 | 7 |
| G535 | 347.316 | -11.567 | 2.275 | 236.963 | 8 |
| G226 | 325.366 | -9.937 | 2.853 | 222.128 | 9 |
| G525 | 320.197 | -12.908 | 3.494 | 218.343 | 10 |
| G124 | 313.891 | -12.304 | 1.428 | 213.930 | 11 |
| G432 | 303.627 | -16.375 | 6.942 | 206.907 | 12 |
| G635 | 298.799 | -10.122 | 1.995 | 203.847 | 13 |
| G321 | 298.215 | -26.498 | 9.550 | 202.403 | 14 |
| G4111 | 295.624 | -21.889 | 7.018 | 200.892 | 15 |
| G125 | 291.561 | -6.656 | 1.898 | 199.221 | 16 |
| G626 | 274.701 | -9.573 | 4.332 | 187.564 | 17 |
| G428 | 274.172 | -21.695 | 6.722 | 186.193 | 18 |
| G133 | 272.323 | -10.900 | 1.795 | 185.619 | 19 |
| G319 | 272.471 | -27.124 | 11.270 | 184.832 | 20 |
| G225 | 268.885 | -15.161 | 5.266 | 183.103 | 21 |
| G522 | 269.855 | -33.397 | 14.405 | 182.658 | 22 |
| G711 | 260.282 | -28.053 | 11.332 | 176.396 | 23 |
| G625 | 256.293 | -3.206 | -0.395 | 175.229 | 24 |
| G513 | 255.293 | -29.638 | 11.931 | 172.868 | 25 |
| G427 | 246.695 | -20.533 | 9.137 | 167.662 | 26 |
| G814 | 238.014 | -10.850 | 4.696 | 162.335 | 27 |
| G622 | 229.065 | -14.949 | 5.526 | 155.865 | 28 |
| G227 | 222.843 | -0.155 | 0.838 | 152.703 | 29 |
| G127 | 218.686 | -15.662 | 6.421 | 148.751 | 30 |
| G435 | 211.283 | 0.827 | -1.575 | 144.703 | 31 |
| G514 | 212.365 | -13.513 | 6.216 | 144.615 | 32 |
| G328 | 211.550 | -10.734 | 5.498 | 144.276 | 33 |
| G318 | 205.929 | -8.754 | 3.304 | 140.457 | 34 |
| G424 | 199.960 | -10.389 | 3.570 | 136.228 | 35 |
| G532 | 194.182 | 13.460 | -4.450 | 134.010 | 36 |
| G634 | 186.710 | -11.299 | 5.584 | 127.211 | 37 |
| G614 | 184.958 | 2.108 | -0.570 | 126.867 | 38 |
| G326 | 183.778 | -6.623 | 2.980 | 125.468 | 39 |
| G732 | 183.128 | -20.951 | 9.859 | 124.128 | 40 |
| G128 | 179.851 | -8.839 | 4.739 | 122.690 | 41 |
| G611 | 178.185 | -16.463 | 8.672 | 121.093 | 42 |
| G327 | 177.542 | -10.770 | 6.215 | 121.028 | 43 |
| G728 | 173.085 | -14.290 | 5.841 | 117.604 | 44 |
| G528 | 172.466 | -13.996 | 7.271 | 117.313 | 45 |
| G636 | 167.301 | -17.649 | 8.254 | 113.490 | 46 |
| G235 | 162.835 | 26.167 | -12.139 | 113.213 | 47 |
| G437 | 165.230 | -9.512 | 5.394 | 112.657 | 48 |
| G312 | 162.712 | 1.218 | 0.095 | 111.590 | 49 |
| G613 | 163.293 | -8.351 | 5.530 | 111.453 | 50 |
| G812 | 161.381 | 0.679 | 3.159 | 110.851 | 51 |
| G811 | 159.422 | 7.434 | -2.513 | 109.752 | 52 |
| G331 | 153.438 | -0.511 | 0.517 | 105.099 | 53 |
| G734 | 149.443 | -0.132 | 3.672 | 102.632 | 54 |
| G533 | 150.189 | -11.367 | 7.737 | 102.344 | 55 |
| G714 | 144.177 | -4.851 | 2.969 | 98.512 | 56 |
| G218 | 142.053 | -1.972 | 2.308 | 97.289 | 57 |
| G727 | 138.855 | -12.341 | 6.145 | 94.368 | 58 |
| G313 | 133.701 | -5.662 | 5.907 | 91.472 | 59 |
| G215 | 118.141 | 4.216 | 0.087 | 81.349 | 60 |
| G712 | 111.126 | -0.333 | 2.311 | 76.263 | 61 |
| G632 | 104.567 | -4.834 | 3.653 | 71.429 | 62 |
| G623 | 103.885 | -4.463 | 3.535 | 70.990 | 63 |
| G821 | 101.311 | 11.100 | -1.783 | 70.356 | 64 |
| G731 | 100.107 | -7.913 | 4.187 | 68.112 | 65 |
| G834 | 98.922 | -8.031 | 4.188 | 67.289 | 66 |
| G633 | 96.246 | 7.395 | -0.400 | 66.626 | 67 |
| G819 | 72.302 | 0.058 | 2.095 | 49.690 | 68 |
| G825 | 71.954 | -5.604 | 5.627 | 49.158 | 69 |
| G733 | 64.180 | -3.267 | 6.084 | 44.095 | 70 |
| G713 | 60.813 | 0.321 | 2.272 | 41.858 | 71 |
| G832 | 49.505 | -2.024 | 2.503 | 33.899 | 72 |
| G422 | 46.816 | 5.246 | 0.749 | 32.638 | 73 |
